# The appropriate frequency and function of decidual Tim-3^+^CTLA-4^+^CD8^+^ T cells are important in maintaining normal pregnancy

**DOI:** 10.1038/s41419-019-1642-x

**Published:** 2019-05-28

**Authors:** Songcun Wang, Fengrun Sun, Mengdie Li, Jinfeng Qian, Chunqin Chen, Mingyan Wang, Xingxing Zang, Dajin Li, Min Yu, Meirong Du

**Affiliations:** 1 Laboratory for Reproductive Immunology, Key Laboratory of Reproduction Regulation of NPFPC, SIPPR, IRD, Shanghai Key Laboratory of Female Reproductive Endocrine Related Diseases, Hospital of Obstetrics and Gynecology, Fudan University Shanghai Medical College, Shanghai, China; 2 Department of Clinical Laboratory, Hospital of Obstetrics and Gynecology, Fudan University, Shanghai, China; 3 Department of Medicine, Montefiore Medical Center, Albert Einstein College of Medicine, Bronx, NY USA; 4 Reproductive Medicine Center, Hospital of Obstetrics and Gynecology, Fudan University Shanghai Medical School, Shanghai, China

**Keywords:** Adaptive immunity, Reproductive disorders

## Abstract

Maternal decidual CD8^+^ T (dCD8^+^ T) cells must integrate the antithetical demands of maternal–fetal tolerance and anti-viral immunity to establish a successful pregnancy. T-cell immunoglobulin mucin-3 (Tim-3) and cytotoxic T-lymphocyte-associated protein 4 (CTLA-4) are two important co-inhibitory molecules that regulating CD8^+^ T cells responses during infection and tumor. In the present study, we examined the co-expression of Tim-3 and CTLA-4 on CD8^+^ T cells during pregnancy and found the higher frequency of Tim-3^+^CTLA-4^+^dCD8^+^ T cells in response to trophoblasts. This Tim-3^+^CTLA-4^+^dCD8^+^ T cells subset showed an active status and produced more anti-inflammatory cytokines. Furthermore, the decreased number and altered function of Tim-3^+^CTLA-4^+^dCD8^+^ T cells correlated to miscarriage. Combined blocking Tim-3 and CTLA-4 pathways were highly effective in inhibiting the production of anti-inflammatory cytokines and were detrimental to the maintenance of pregnancy. Together, these findings supported that Tim-3 and CTLA-4 pathways might play positive roles in the establishment and/or maintenance of maternal–fetal tolerance so to promote the maintenance of normal pregnancy. So the reproductive safety must be considered, especially when anti-Tim-3/CTLA-4 antibody (and other immune checkpoint inhibitors) are used in pregnancy.

## Introduction

During a successful pregnancy in placental mammals, the balance between immune tolerance allowing allogeneic fetal trophoblasts (Tros) to invade maternal tissues and immune defense against a variety of pathogens is required to be established. Disruption of this immune balance is believed to be associated with a lot of pregnancy-related complications, such as recurrent spontaneous abortion (RSA), pre-eclampsia, and fetal intrauterine growth restriction^1^. For many years, the placenta has been regarded as a pseudo-malignant type of tissue or a physiological metastasis^2^, so the understanding of the mechanisms that underlie the immune regulation during normal pregnancy not only provides insights into the etiology of pregnancy complications, but also may impact on studies of tumor immunological tolerance.

The maternal immune cells, contacting with Tros, make important contributions to the maintenance of successful pregnancy. Decidual CD8^+^ T (dCD8^+^ T) cells form the largest fraction of decidual T cells and are the main candidates to recognize and respond to human leukocyte antigen C (HLA-C) expressed by Tros^3^. Upon in vitro stimulation, dCD8^+^ T cells degranulated, proliferated, and produced IFN-γ, TNF-α, perforin, and granzymes^4^. The key question is how dCD8^+^ T cells elicit cytolytic responses to pathogens while render comparatively dysfunctional or impaired functions to fetal antigens. dCD8^+^ T cells were observed to contain CD8-Treg and effector-memory T-cell subsets, and display enhanced functionality in terms of degranulation and cytokine production on a per-cell basis^5^. Gene-expression analysis of effector-memory dCD8^+^ T cells demonstrated a mixed transcriptional signature of T-cell dysfunction, activation, and effector function^6^. High-protein expression of co-inhibitory molecules was observed on effector-memory dCD8^+^ T cells^6,7^. These co-inhibitory molecules, including T-cell immunoglobulin mucin-3 (Tim-3) and cytotoxic T-lymphocyte-associated protein 4 (CTLA-4), have been implicated to identify dysfunctional T cells, as progressive increase in the diversity and amount of co-inhibitory molecules expressed on T cells leads to the dysfunction of T cells^8,9^. Blockade of these inhibitory receptors to improve T-cell responses is considered as a novel strategy for the treatment of chronic infection and some tumors^10,11^.

In the present study, we aimed not only to determine the co-expression of Tim-3 and CTLA-4 on dCD8^+^ T cells, but also to investigate their contributions to the maternal–fetal immunity in early pregnancy. Our findings showed that Tim-3 and CTLA-4 co-expression on dCD8^+^ T cells linked to immunosuppressive phenotype. Altered frequency and function of Tim-3^+^CTLA-4^+^dCD8^+^ T cells were associated with miscarriage. Furthermore, in animal studies, anti-Tim-3/CTLA-4 antibody clearly increased the risks of abortions. The current data demonstrated that Tim-3 and CTLA-4 signal pathways played vital roles in the establishment and/or maintenance of maternal–fetal tolerance by affecting the phenotype and functions of dCD8^+^ T cells. So the reproductive safety must be considered, though immune checkpoints inhibitors seem to be effective on the therapy of some diseases, especially when they are used in pregnancy.

## Results

### Tros contributed to the higher proportion of Tim-3^+^CTLA-4^+^CD8^+^ T cells at the maternal–fetal interface

**Fig. 1:**
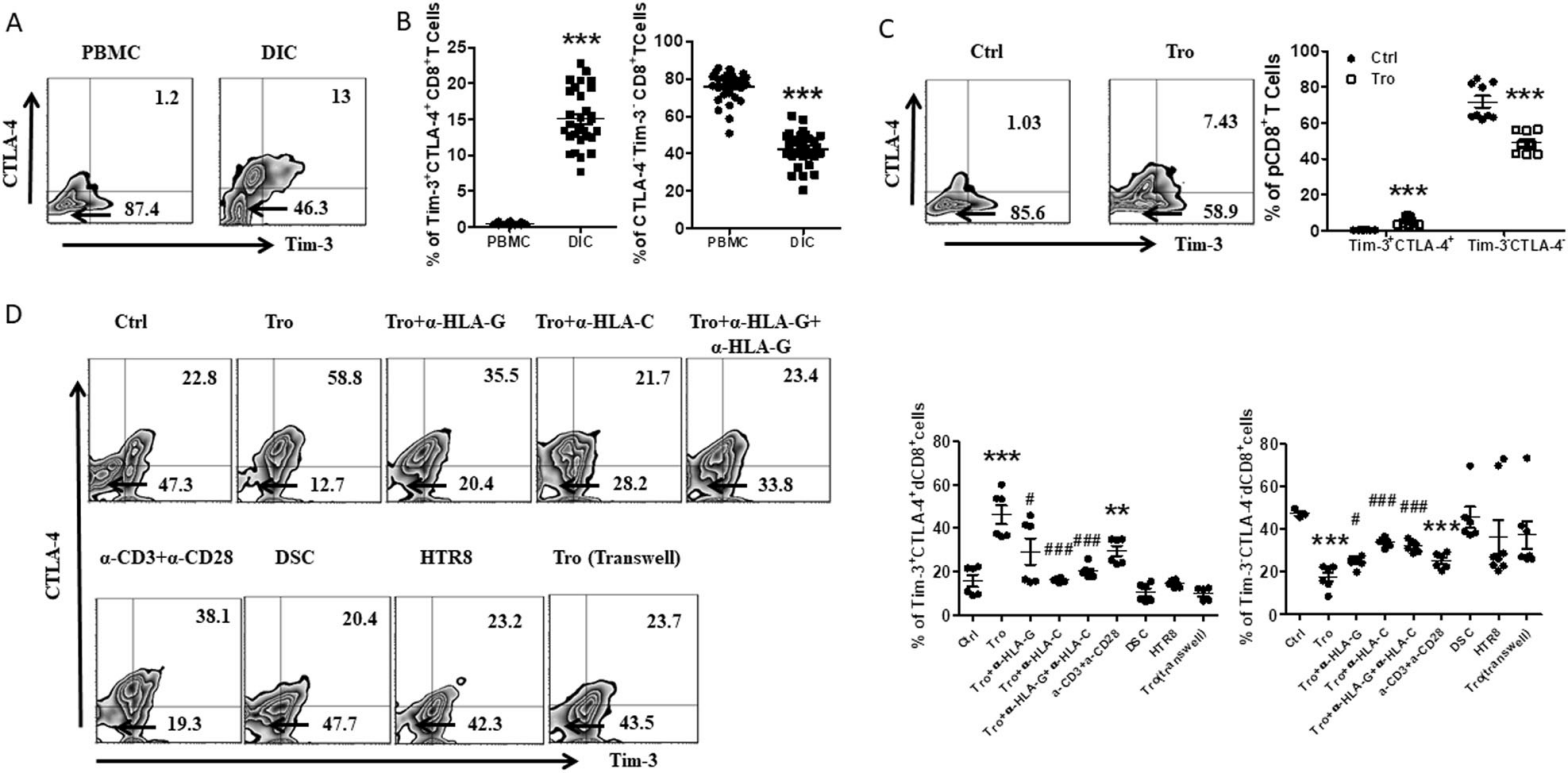
Co-expression of Tim-3 and CTLA-4 on CD8^+^ T cells during human early pregnancy. **a**, **b** Flow cytometric analysis (**a**) and quantification (**b**) of frequency of Tim-3 and CTLA-4 co-expression on gated CD8^+^ T cells from peripheral blood mononuclear cells (PBMCs) and decidual immune cells (DICs) during human first-trimester pregnancy (*n* = 30). ***p < 0.001. **c** Quantification of flow cytometric analysis of Tim-3 and CTLA-4 co-expression on peripheral CD8^+^ T (pCD8^+^ T) cells with or without co-culture with trophoblasts (Tros) for 48 h. *n* = 9. ***p < 0.001. **d** Quantification of flow cytometric analysis of Tim-3 and CTLA-4 expression on decidual CD8^+^ T (dCD8^+^ T) cells cultured alone or co-cultured with equal numbers of Tros (directly or indirectly), or decidual stromal cells (DSCs), or human HTR8/SVneo cells. The αHLA-G/HLA-C, αCD3/CD28 antibodies were used in some wells. **p < 0.01, ***p < 0.001, compared with the control. ^#^*p* < 0.5, ^###^*p* < 0.001, compared with the group co-cultured with Tros. Data represent the mean ± standard error of the mean (SEM). The flow cytometry plots are representative of three independent experiments

We first compared the expression levels of Tim-3 and CTLA-4 on CD8^+^ T cells from paired decidua and peripheral blood freshly isolated from clinically normal first-trimester pregnancies (terminated for nonmedical reasons). Tim-3 and CTLA-4 were expressed on significantly higher proportions of dCD8^+^ T cells than in peripheral CD8^+^T (pCD8^+^ T) cells, as the Tim-3^+^CTLA-4^+^ cells comprised about 14% of dCD8^+^ T cells, while only 1% of pCD8^+^ T cells. In the contrast, 80% of the pCD8^+^ T cells, but only 40% of the dCD8^+^ T cells were Tim-3^−^CTLA-4^−^ (Fig. 1a, b).

Then why are Tim-3 and CTLA-4 highly expressed on dCD8^+^ T cells? During a successful pregnancy in placental mammals, the endometrium goes through decidualization upon embryo implantation to form the decidua, and Tros invades into the decidua^1^. Due to the invasive property, high proliferation and immune escape capacity, the placenta is regarded as a pseudo-malignant type of tissue^2^. As tumor microenvironment is responsible to the higher expression of co-inhibitory receptors on tumor-infiltrating T cells^12^, we first established a co-culture system of Tros and dCD8^+^ T cells or pCD8^+^ T cells. As shown in Fig. 1c, d, after 48 h, both in pCD8^+^ T and dCD8^+^ T cells, the frequency of Tim-3^+^CTLA-4^+^ cells increased, while Tim-3^-^CTLA-4^−^ cells decreased. But co-culture with DSCs (the predominant cell type of the maternal decidua) or HTR8/Svneo (an immortalized human extravillioustrophoblast cell line) had no effect on the expression of Tim-3 and CTLA-4 on dCD8^+^ T cells.

Tros express HLA-G and HLA-C that are associated with immune tolerance^4^, and directly contact with dCD8^+^ T cells. We further added anti-HLA-G or/and anti-HLA-C antibody into the co-culture system, and found the inhibition of Tros-induced up-regulation of Tim-3 and CTLA-4 co-expression on dCD8^+^ T cells (Fig. 1d). When a trans-well insert was used to separate Tros and dCD8^+^ T cells, the co-expression of Tim-3/CTLA-4 on dCD8^+^ T cells no longer changed. As activation with anti-CD3/CD28 antibody was also sufficient to increase Tim-3/CTLA-4 co-expression on dCD8^+^ T cells, we speculated that Tros contributed to the higher Tim-3/CTLA-4 co-expression at the maternal–fetal interface by activating dCD8^+^ T cells in a direct contact.

### dCD8^+^ T cells co-expressing Tim-3/CTLA-4 displayed an activated status

**Fig. 2:**
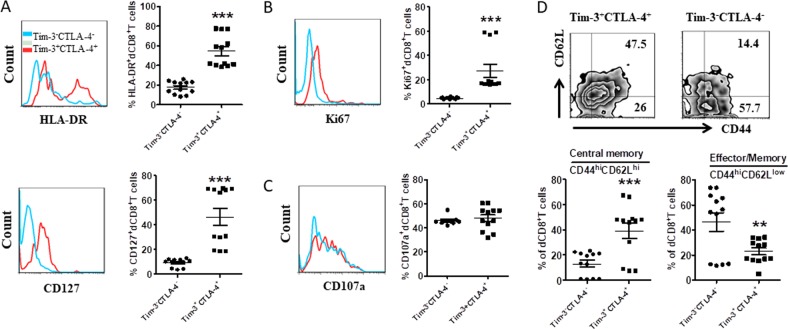
Characterization of Tim-3^+^CTLA-4^+^dCD8^+^ T cells during human early pregnancy. **a** Expression of HAL-DR and CD127 on Tim-3^+^CTLA-4^+^ and Tim-3^−^CTLA-4^−^dCD8^+^ T cells from the first trimester of normal pregnancy (*n* = 12). **b** Quantification of Ki67 staining in Tim-3^+^CTLA-4^+^ and Tim-3^-^CTLA-4^−^dCD8^+^ T cells from the first trimester of normal pregnancy (*n* = 12). **c** Expression of CD107a on Tim-3^+^CTLA-4^+^ and Tim-3^−^CTLA-4^−^dCD8^+^ T cells from the first trimester of normal pregnancy (*n* = 11). **d** DICs from the first trimester of pregnancy (*n* = 9) were stained with antibodies against CD8, CD44, CD62L, Tim-3, and CTLA-4, and then analyzed by flow cytometry. *n* = 9. Data represent the mean ± SEM. **p < 0.01, ***p < 0.001. A representative dot plot is also shown

Given the increased expression of Tim-3/CTLA-4, Tim-3^+^CTLA-4^+^dCD8^+^ T cells during human early pregnancy could be dysfunctional. Alternatively, expression of inhibitory receptors could be the consequence of local activation of CD8^+^ T cells. To study these two possibilities, we examined the activation extent of Tim-3^+^CTLA-4^+^ and Tim-3^-^CTLA-4^−^dCD8^+^ T cells by using the activation markers HLA-DR and CD127. Tim-3^+^CTLA-4^+^dCD8^+^ T cells were significantly more activated than Tim-3^−^CTLA-4^−^dCD8^+^ T cells in both populations (Fig. 2a). As the expression of CD127 is crucial for T-cell survival through homeostatic proliferation^13^, we next compared the proliferation of Tim-3^+^CTLA-4^+^ and Tim-3^−^CTLA-4^−^dCD8^+^ T cells, and found that Tim-3^+^CTLA-4^+^dCD8^+^ T cells actually had the stronger ability to proliferate (Fig. 2b). Despite their activated status, the expression of CD107a, a lysosome-associated membrane glycoprotein, was comparable in Tim-3^+^CTLA-4^+^dCD8^+^ T cells compared with the negative cells (Fig. 2c).

It has been reported that CD127-expressing CD8^+^ T cells have increased memory potential^14^, we next analyzed Tim-3 and CTLA-4 expression on different memory T-cell subsets during pregnancy. Flow cytometric analysis demonstrated that most central memory cells (T_CM_, CD44^hi^CD62L^hi^) were Tim-3^+^CTLA-4^+^, while effector-memory cells (T_EM_, CD44^hi^CD62L^low^)^15^ were primarily Tim-3^−^CTLA-4^−^ (Fig. 2d).

**Fig. 3:**
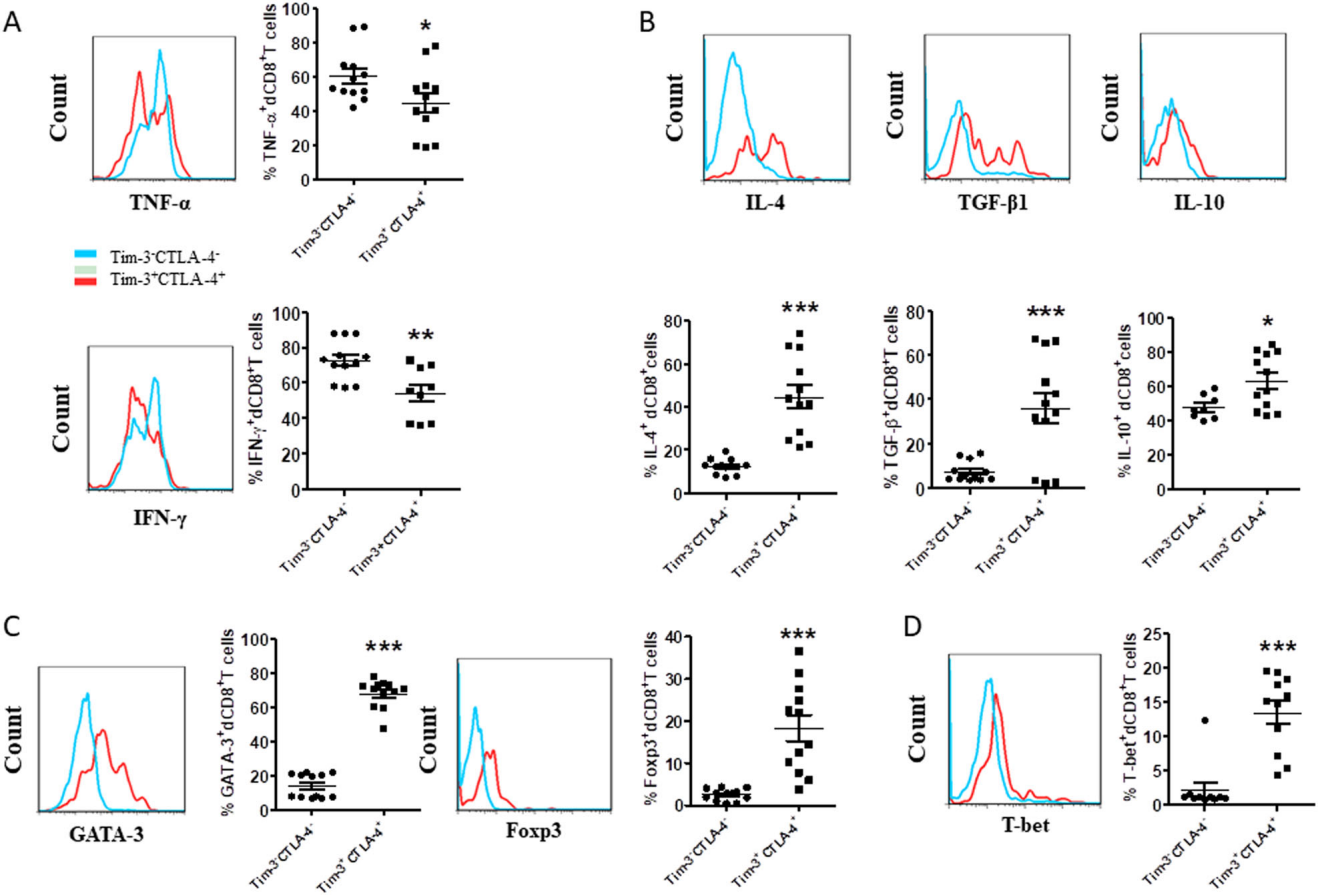
Cytokines production in dCD8^+^ T cells during normal pregnancy. **a** Expression of the Th1-type cytokines TNF-α and IFN-γ in Tim-3^+^CTLA-4^+^, and Tim-3^-^CTLA-4^−^dCD8^+^ T cells from the first trimester of normal pregnancy. A representative flow cytometry plot (left) and quantitation (right) are shown. *n* = 9. **b** Quantitation of flow cytometric analysis of IL-4, TGF-β1, and IL-10 of Tim-3^+^CTLA-4^+^, and Tim-3^−^CTLA-4^−^dCD8^+^ T cells. *n* = 12. **c**, **d** Expression of GATA-3, Foxp3 (**c**) and T-bet (**d**) of Tim-3^+^CTLA-4^+^, and Tim-3^−^CTLA-4^−^dCD8^+^ T cells. *n* = 12. Data represent the mean ± SEM. The flow cytometry plots are representative of three independent experiments. *p < 0.05, **p < 0.01, ***p < 0.001, compared with Tim-3^−^CTLA-4^−^dCD8^+^ T cells

Furthermore, we stimulated dCD8^+^ T cells with phorbol 12-myristate 13-acetate (PMA) and ionomycin to assess effector cytokine production. After exposure to PMA and ionomycin for 4 h, the percentage of TNF-α and IFN-γ producing cells in Tim-3^+^CTLA-4^+^dCD8^+^ T cells were reduced compared with Tim-3^-^CTLA-4^−^dCD8^+^ T cells (Fig. 3a). But the production of immune-regulatory cytokines, including IL-4, TGF-β1, and IL-10 were significantly increased by Tim-3^+^CTLA-4^+^dCD8^+^ T cells (Fig. 3b). In accordance with this, the expression of GATA-3 (Th2-type transcription factor) and Foxp-3 (Treg-type transcription factor) were also higher in the double positive subpopulation. Interestingly, T-bet (Th1-type transcription factor) expression was also increased on Tim-3^+^CTLA-4^+^dCD8^+^T cells. Whether there were some post-translational modifications that blocked translation of TNF-α and IFN-γ, or other transcription factors that induced these two cytokines translation in Tim-3^+^CTLA-4^+^dCD8^+^ T cells, needed further study. Together, at least, these data indicated that dCD8^+^ T cells that co-expressed Tim-3 and CTLA-4 were activated and their functionality were not weakened.

### Altered frequency and phenotype of Tim-3^+^CTLA-4^+^dCD8^+^ T cells in RSA

**Fig. 4:**
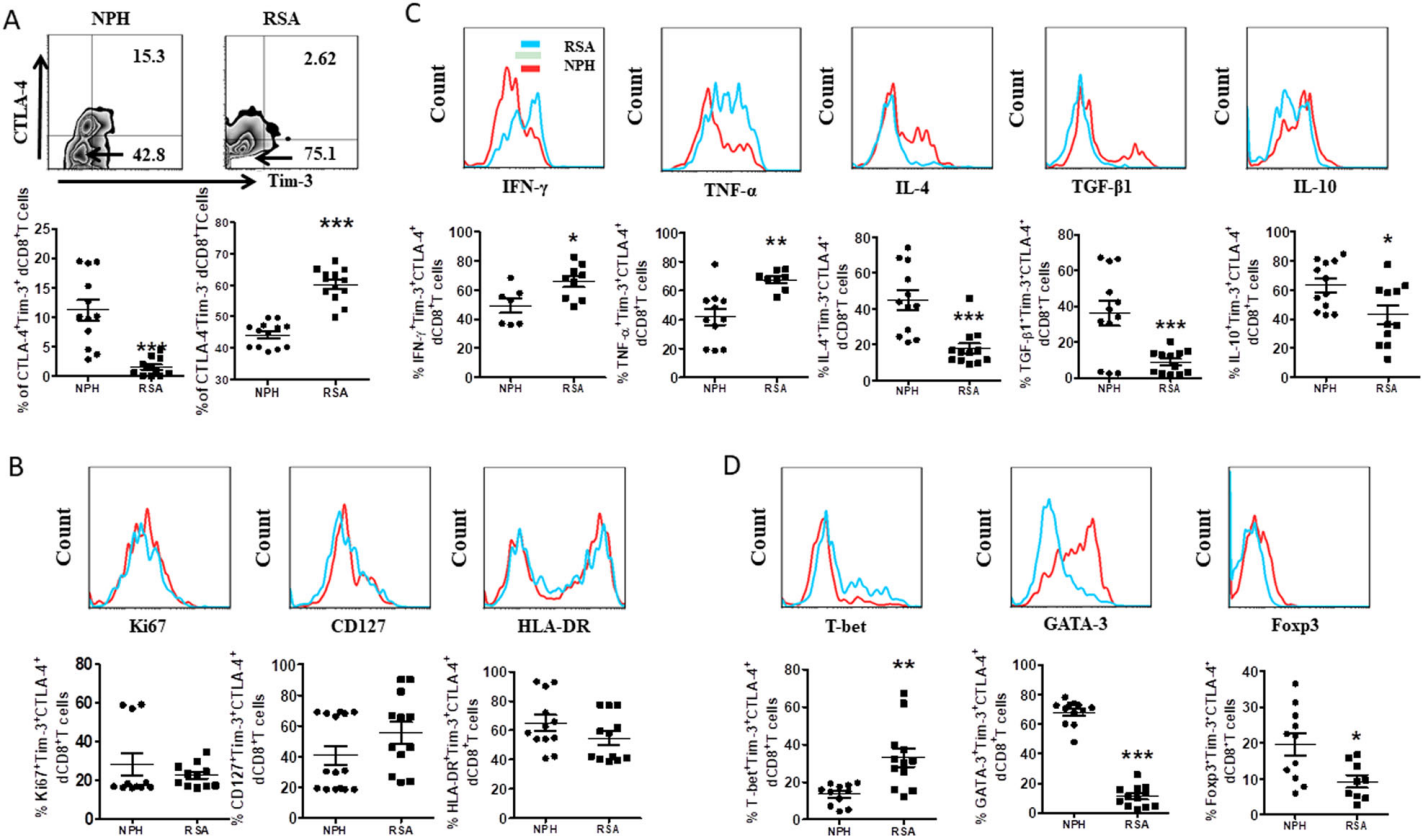
Decreased cell number of Tim-3^+^CTLA-4^+^dCD8^+^ T cells with deficient function in patients of recurrent spontaneous abortion. **a** Frequency of Tim-3 and CTLA-4 co-expressing cells in gated CD8^+^ T cells from DICs from normal pregnant subjects (NPH, normal pregnancy of human, *n* = 23) and patients who diagnosed as recurrent spontaneous abortion (RSA, *n* = 27) as determined by flow cytometric analysis. A representative dot plot is also shown. **b** Expression of ki67, CD127, and HLA-DR of Tim-3^+^CTLA-4^+^dCD8^+^ T cells from normal pregnancy and RSA. **c**, **d** Cytokines production and transcription factor expression onTim-3^+^CTLA-4^+^dCD8^+^ T cells from normal pregnancy and RSA was assessed by flow cytometric analysis. Data represent the mean ± SEM. *p < 0.05, **p < 0.01, ***p < 0.001. NPH normal pregnancy of human, RSA recurrent spontaneous abortion

Next, we compared the frequency and phenotype of Tim-3^+^CTLA-4^+^dCD8^+^ T cells from normal pregnancy and RSA patients. We found that there is an increased proportion of dCD8^+^ T cell in RSA patients (data not shown). As shown in Fig. 4a, the co-expression of Tim-3 and CTLA-4 on dCD8^+^ T was much lower, while the number of Tim-3^−^CTLA-4^−^dCD8^+^ T cells was higher in RSA patients than that form normal pregnancy. Apart from the decreased frequency, the expression of ki67, CD127, and HLA-DR by Tim-3^+^CTLA-4^+^dCD8^+^ T cells were comparable compared with Tim-3^−^CTLA-4^−^dCD8^+^ T cells in RSA patients (Fig. 4b). Furthermore, in RSA patients, Tim-3^+^CTLA-4^+^dCD8^+^ T cells produced more TNF-α and IFN-γ, but less IL-4, TGF-β1 and IL-10 (Fig. 4c). The expression of T-bet was also increased on Tim-3^+^CTLA-4^+^dCD8^+^ T cells, while GATA-3 and Foxp3 were decreased in RSA patients (Fig. 4d).

**Fig. 5:**
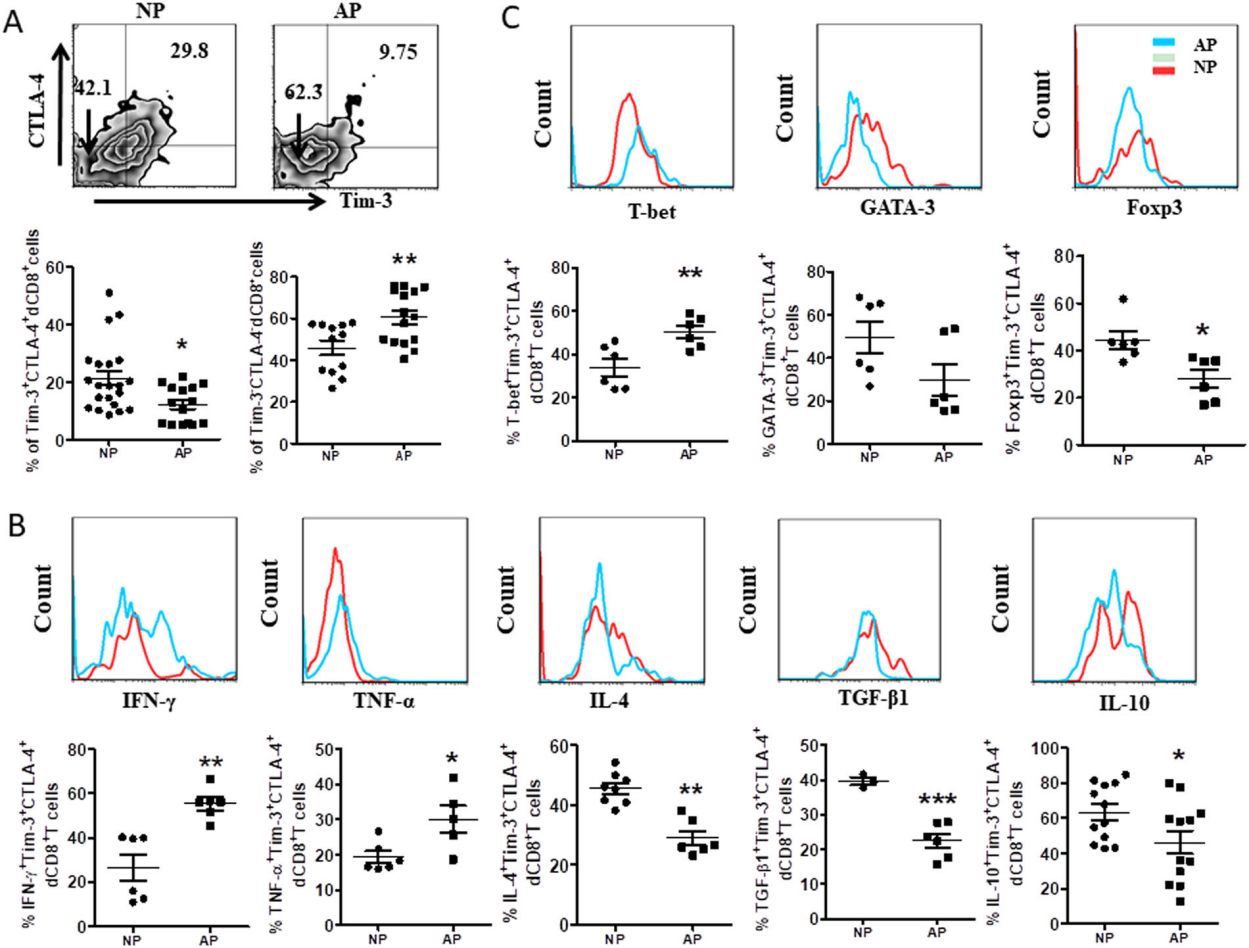
Altered frequency and function of Tim-3^+^CTLA-4^+^dCD8^+^ T cells in early mouse pregnancy loss. **a** Frequency of Tim-3 and CTLA-4 co-expressing cells in gated CD8^+^ T cells from DICs of normal pregnant (NP) and abortion-prone (AP) mice. A representative dot plot is also shown. **b**, **c** Cytokines production and transcription factors expression by Tim-3^+^CTLA-4^+^dCD8^+^ T cells from NP and AP mice assessed by flow cytometric analysis. Data represent the mean ± SEM. *p < 0.05, **p < 0.01, ***p < 0.001. NP normal pregnancy, *n* = 10; AP abortion prone, *n* = 13. Data are representative of 4–5 independent analyses. *p < 0.05, **p < 0.01, ***p < 0.001

But lower IL-17 and higher ROR-γt expression were observed in Tim-3^+^CTLA-4^+^dCD8^+^ T cells from RSA patients than that from normal pregnancy (Fig. S1). As Th17 cells were important in the maintenance of normal pregnancy^16^, the possible mechanism and consequences of this unique Th17-type cytokines pattern in Tim-3^+^CTLA-4^+^dCD8^+^ T cells during RSA needed further study. We also established an abortion-prone model by female CBA/J × male DBA/2 mice, and observed similar phenomenon that decreased number and disordered function of Tim-3^+^CTLA-4^+^dCD8^+^ T cells in miscarriage (Fig. 5).

### Combining Tim-3 blockade with CTLA-4 affected dCD8^+^ T cells functions during normal pregnancy

**Fig. 6:**
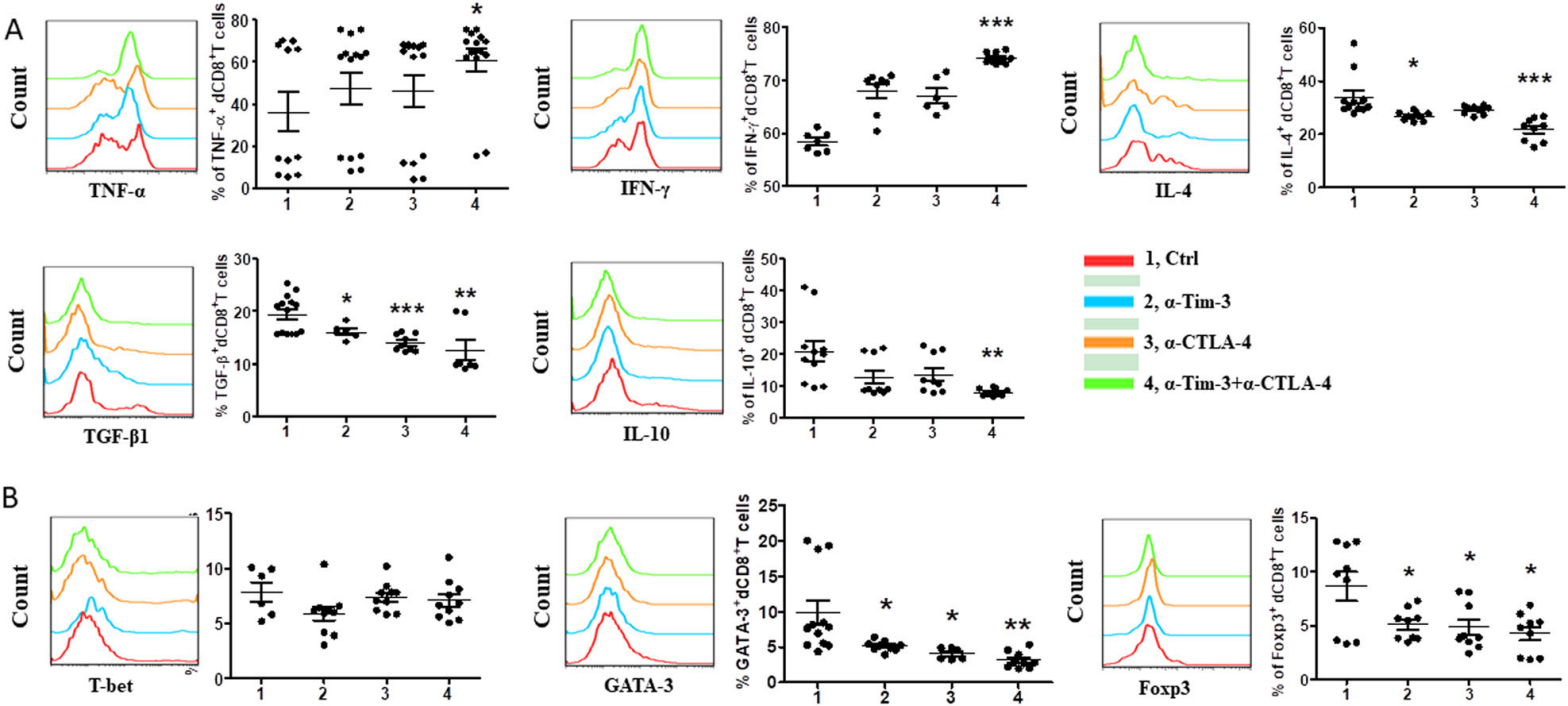
Effect of blocking Tim-3 and CTLA-4 signaling pathways on human dCD8^+^ T cells function. **a** Expression of Th1-, Th2-, and Treg-type cytokines of dCD8^+^ T cells cultured for 48 h in the presence or absence of anti-Tim-3 antibody (10 μg/ml), anti-CTLA-4 antibody (10 μg/ml), or both. (**b**) Representative images and quantification of flow cytometric analyses of transcription factors expression by dCD8^+^ T cells following treatment with the indicated blocking antibodies. Data represent the mean ± SEM. *n* = 12. *p < 0.05, **p < 0.01, ***p < 0.001, compared with the control group

As the frequency and function of Tim-3^+^CTLA-4^+^dCD8^+^ T cells were both altered in miscarriage, we further tested whether blocking the Tim-3 and CTLA-4 pathways could change the functionalities of dCD8^+^ T cells. In the first assay, we stimulated dCD8^+^ T cells with anti-CD3/CD28 antibodies in the presence or absence of antibodies blocking Tim-3/CTLA-4 pathway, respectively or combinations of blocking antibodies. After 48 h, expression levels of intracellular cytokines and transcription factors in dCD8^+^ T cells were analyzed. Compared with the control group, combinations of blocking Tim-3 and CTLA-4 increased the proinflammatory TNF-α and IFN-γ, but decreased anti-inflammatory IL-4, TGF-β1 and IL-10 production of dCD8^+^ T cells (Fig. 6a). Anti-Tim-3 or anti-CTLA-4 antibody alone was insufficiently to affect all the cytokines production. As shown in Fig. 6b, the expression of GATA-3 and Foxp3 by dCD8^+^ T cells were downregulated following single or combined antibody blockade. But Tim-3 or CTLA-4 blockade had no effect on IL-17A production and T-bet and ROR-γt expression by CD8^+^ T cells at the maternal–fetal interface (Fig. 6b and Fig. S2).

**Fig. 7:**
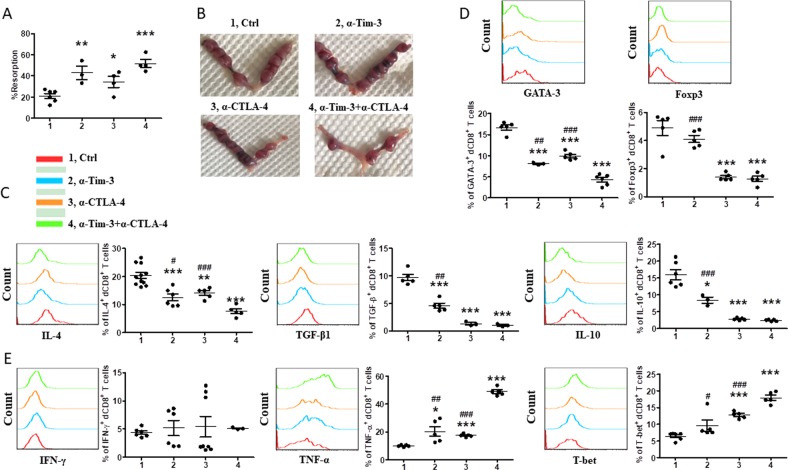
In vivo roles of targeting Tim-3 or/and CTLA-4 pathways during early pregnancy. **a** The percent of fetal resorption of pregnant CBA/J females treated with isotype IgG, anti-Tim-3 antibody, anti-CTLA-4 antibody, or both antibodies i.p. at doses of 500, 250, and 250 mg at days 4.5, 6.5, and 8.5, respectively. **b** Representative images of embryos from pregnant CBA/J females following treatment with the indicated blocking antibodies. **c**–**e** Quantification of flow cytometric analysis of cytokines production and transcription factors expression by dCD8^+^ T cells pregnant CBA/J females following treatment with the indicated blocking antibodies. Data represent the mean ± SEM of *n* = 5–12 mice per group and are representative of four independent analyses. *p < 0.05, **p < 0.01, ***p < 0.001, compared with the control group. ^#^*p* < 0.5, ^##^*p* < 0.01, ^###^*p* < 0.001, compared with the group of anti-Tim-3 and anti-CTLA-4

In the second assay, we examined pregnant CBA/J females challenged with Tim-3- and/or CTLA-4-blocking antibody. Treatment with either blocking antibody caused greater susceptibility to fetal loss (Fig. 7a, b). Furthermore, dual blockade of the Tim-3 and CTLA-4 pathways had a combined effect, leading to the highest rate of embryo resorption (Fig. 7a, b). These data indicated that Tim-3 and CTLA-4 might play a protective role during successful pregnancy in vivo.

Does the more fetal loss of treated mice result from the dysfunction of dCD8^+^ T cells under anti-Tim-3 or/and anti-CTLA-4 antibody treatment? Analysis of the dCD8^+^ T cells from the treated mice revealed that IL-4, TGF-β1, and IL-10 production, and GATA-3 and Foxp-3 expression of dCD8^+^ T cells were decreased. And treatment with both antibodies combined exerted the greatest effect on these cytokines production (Fig. 7c, d). Treatment with anti-Tim-3 and/or anti-CTLA-4 also significantly enhanced TNF-α production and T-bet expression, but the production of IFN-γ by dCD8^+^ T cells was not affected (Fig. 7e). Taken together with our in vitro data, Tim-3 and CTLA-4 pathways regulated immune responses in dCD8^+^ T cells so to play important role in the maintenance of normal pregnancy.

## Discussion

For many years, it has been believed that a shift in the maternal immune response towards a Th2 and Treg bias of CD4^+^ T cells at the maternal–fetal interface is crucial for maintaining a successful pregnancy^1,17^. And also, it has been accepted that maternal adaptation to the semi allogeneic fetus is more complicated than this initial concept implied during normal pregnancy^18^. CD8^+^ T cells are the most dominant T cells at the maternal–fetal interface. Though HLA-C expressed by Tros can serve as targets for CD8^+^ T cells^19^, there is no evidence that CD8^+^ T-cell responses compromise pregnancy outcome^20^. In recent years, suppressor or regulatory CD8^+^ T cells have been proposed to contribute to maternal–fetal tolerance^19,21^. In this paper, we found Tim-3 and CTLA-4 were expressed in significantly higher proportions of dCD8^+^ T cells than pCD8^+^ T cells, and this Tim-3^+^CTLA-4^+^dCD8^+^ T cells subset might play important roles in the maintenance of normal pregnancy.

Besides the expression of highly polymorphic HLA-C molecules, Tros also express the nonpolymorphic HLA-G. These two molecules are the main molecules which are needed for immune tolerance establishment^4^. HLA-G^+^ Tros were also shown to have the ability to increase the number of Treg cells at the maternal–fetal interface^22^. We found that Tros contributed to promote Tim-3^+^CTLA-4^+^dCD8^+^ T cells expansion depending on HLA-C/G. Previous studies, including ours, have revealed that Tros have the unique ability to instruct decidual immune cells to develop a regulatory phenotype for fetal tolerance^23,24^. DSCs had no effect on the co-expression of dCD8^+^ T cells, further confirming the importance of Tros in the establishment of maternal–fetal tolerance. Importantly, HTR8/SVneo cell line, which is widely used as a substitute for human primary extravillious tros, could not induce the expression of Tim-3 and CTLA-4 on dCD8^+^ T cells. Though HTR8/SVneo cells have been proven effective for recapitulating key aspects of extravillious tros, there exit many differences between them. For instance, HTR-8/SVneo cells lack IL-10 production, only partially induce the regulatory cells, and, contrary to placental tissue, enhance T help cell activation^24^. Thus, questions remain regarding the validity of using immortalized cell lines to represent the in vivo environment.

T cells co-expressing inhibitory receptors are always believed to be the most severely exhausted T-cell subset^25,26^. In contrast, we found the co-expression of Tim-3 and CTLA-4 on dCD8^+^ T cells defined a more active status of CD8^+^ T cells, as evidenced by stronger proliferation and higher expression of activation markers. A recent study also reported that tumor-infiltrating T cells that expressed inhibitory receptors were activated^27^. Most of this activated subset of dCD8^+^ T cells (Tim-3^+^CTLA-4^+^dCD8^+^ T cells) was central memory cells, though most dCD8^+^ T cells were thought to have an effector-memory phenotype^5,7,28^. Actually, a significant proportion of effector-memory cells did exhibit a Tim-3^−^CTLA-4^−^ phenotype, as most dCD8^+^ T cells were Tim-3^−^CTLA-4^−^. CD44^+^CD62L^+^ T_CM_ is less cytolytic compared to CD44^+^CD62L^−^ T_EM_, exhibiting increased survival with a capacity of antigen-independent self-renewal^29^. This was also parallel with our results that a majority of central memory cells were Tim-3^+^CTLA-4^+^ at the maternal–fetal interface, and this subset possessed higher proliferative capability and produced more anti-inflammatory or regulatory cytokines to further promote maternal–fetal tolerance.

We observed decreased frequency and disordered function of Tim-3^+^CTLA-4^+^dCD8^+^ T cells in RSA patients and abortion-prone mouse models. But are the altered frequency and function of Tim-3^+^CTLA-4^+^dCD8^+^ T cells the cause or the consequence of the failure of pregnancy? Combination of Tim-3 and CTLA-4 blockade resulted in the decreased production of anti-inflammatory cytokines of dCD8^+^ T cells both in vivo and in vitro. Furthermore, pregnant CBA/J females treated with Tim-3 and/or CTLA-4 blocking antibodies became more susceptible to fetal loss. These data indicated that the reduction in proportion and the abnormity in functionality were more likely to be the reason, at least one of the reasons, leading to the development of miscarriage.

Of note, in this study, the production of proinflammatory cytokines of Tim-3^+^CTLA-4^+^dCD8^+^ T cells were lower than that of Tim-3^-^CTLA-4^−^dCD8^+^ T cells, while T-bet^+^ cells in Tim-3^+^CTLA-4^+^dCD8^+^ T cells were more than that in Tim-3^−^CTLA-4^−^dCD8^+^ T cells. Furthermore, anti-Tim-3 and/or anti-CTLA-4 antibodies were not always restored the production of proinflammatory cytokines and transcription factors as expected. Whether it is the maternal–fetal microenvironment that shapes distinct signaling pathways of Tim-3 and CTLA-4 in dCD8^+^ T cells, and what differentiates CD8^+^ T cells in this specific microenvironment, remained to be determined. For example, the progesterone produced by placenta could increase the production of IL-4 by dCD8^+^ T cells, but also decrease the production of both proinflammatory IFN-γ and TNF-α and anti-inflammatory IL-10 and IL-5^30^. Whether steroid hormone produced by the placenta during pregnancy, or other factors influence the effects of anti-Tim-3/CTLA-4 antibodies on dCD8^+^ T cells need further researches.

**Fig. 8:**
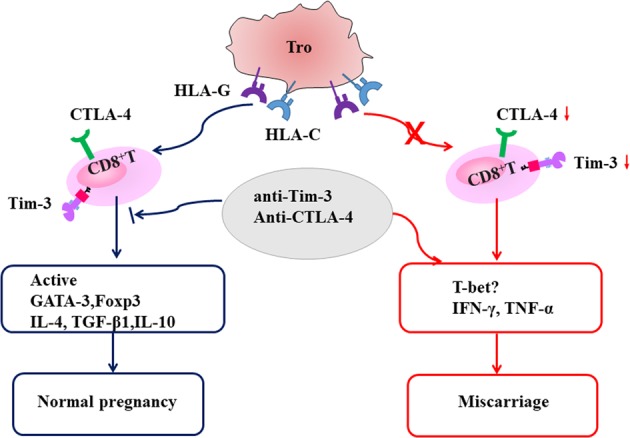
Schematic diagram of Tim-3 and CTLA-4 signals in regulating maternal–fetal interface. Tros contributed to the higher co-expression of Tim-3 and CTLA-4 on dCD8^+^ T cells during normal pregnancy in HLA-C/G dependent manner, and decreased Tim-3 and CTLA-4 expression on dCD8^+^ T cells might be associated with miscarriage because of the dysfunction of dCD8^+^ T cells. The Tim-3^+^CTLA-4^+^dCD8^+^ T cells displayed an active status and produced more anti-inflammatory or regulatory cytokines. Blockade Tim-3 and CTLA-4 pathways leaded to the disorder of dCD8^+^ T cells function and more fetal loss. The expression of TNF-α and IFN-γ on dCD8^+^ T cells according to the Tim-3 and CTLA-4 signaling pathways seemed to be uncertainly dependent on T-bet. Whether there were some post-translational modifications that blocked translation of TNF-α and IFN-γ, or other transcription factors (beside T-bet) that induced these two cytokines translation inTim-3^+^CTLA-4^+^dCD8^+^ T cells, needed further study

In summary (Fig. 8), we concluded the higher co-expression of Tim-3 and CTLA-4 on dCD8^+^ T cells during normal pregnancy, and decreased Tim-3 and CTLA-4 on dCD8^+^ T cells might be associated with miscarriage. The Tim-3^+^CTLA-4^+^dCD8^+^ T cells displayed an active status and produced more anti-inflammatory cytokines. Blockade of Tim-3 and CTLA-4 pathways leaded to the dysfunction of dCD8^+^ T cells and more fetal loss. Tim-3 and CTLA-4 might be promising early warming targets of RSA. Furthermore, antibodies that block the immune inhibitory pathways, such as CTLA-4, PD-1, Tim-3 and so on, have provided a major treatment advance in patients with cancers and chronic infections^27,31,32^, according to our data, the reproductive safety must be a criterion considered in the assessment of immuno-therapeutic agents.

## Materials and methods

### Ethical approval

This study was approved by the Research Ethics Committee of the Obstetrics and Gynecology Hospital, Fudan University (No. Kyy2017-50). Every participant signed a written informed consent form. All of animals were conducted in accordance with the National Guidelines for Animal Care and Use in Research (China). The experimental methods in particular were carried out in accordance with the approved guidelines.

### Human samples

Whole-peripheral blood, villous, and decidual tissues of human first-trimester pregnancies were obtained from clinically normal pregnancies (terminated for nonmedical reasons, had at least one successful pregnancy and no history of spontaneous abortions, *N* = 69) and miscarriages (diagnosed as RSA, and excluding those resulting from endocrine, anatomic, genetic abnormalities, infection, etc., *N* = 27). The clinical characteristics of enrolled subjects are summarized in Table 1. Samples were immediately collected for the isolation of peripheral blood mononuclear cells (PBMCs), trophoblasts, decidual stromal cells (DSCs), and decidual immune cells (DICs).

**Table 1:** Clinical characteristics of enrolled subjects

Subjects	NPH	RSA	*p*
Number	69	27	ns
Age mean (years)^a^	28.07 ± 0.86	28.96 ± 0.98	ns
Age range (years)	20–39	22–38	ns
Previous spontaneous abortion (number)^a^	–	2.92 ± 0.22	–
Pregnancy week (samples were collected)^a^	6.26 ± 0.11	6.28 ± 0.09	ns
Treatment history	–	–	–

Normal early pregnant women (NPH); patients undergoing spontaneous abortion who also had a history of two or more consecutive spontaneous abortions before 20 weeks gestation without known causes (RSA).

^a^Median ± standard error of the mean (SEM).

### Human cell isolation

PBMCs were isolated from peripheral blood samples of normal pregnancies using Ficoll density gradient centrifugation (Huajing, China).

Trophoblasts were isolated by trypsin-DNase I (Applichem, Germany) digestion and discontinuous Percoll gradient centrifugation from the placenta tissues as described previously^33^.

DICs and DSCs were obtained from the normal or RSA decidual tissue digesting in RPMI 1640 (HyClone, USA) supplemented with collagenase type IV (1.0 mg/ml, CLS-1, Worthington Biomedical, USA) and DNase I (150 U/ml, Applichem, Germany) as described previously^33^.

CD8^+^ T cells were isolated by magnetic affinity cell sorting using CD8 microbeads (MiltenyiBiotec, Germany).

### Co-culture of trophoblasts and dCD8^+^ T cells

Freshly isolated trophoblasts were seeded at a density of 2 × 10^5^ cells/ml per well in Matrigel (Coring, USA)-coated 24-well plates overnight. The cells were then washed twice with phosphate-buffered saline (PBS, HyClone, USA). Equal numbers of dCD8^+^ T cells or pCD8^+^ T cells were added to each well. In some wells, anti-HLA-C (10 μg/ml, clone W6/32; Biolegend, U.S.A), HLA-G (10 μg/ml, clone 87G; Biolegend, USA) were added. dCD8^+^T cells were also cultured with plate-bound anti-CD3 antibody (OKT-3; 5 μg/ml, Biolegend, USA) plus soluble anti-CD28 antibody (28.2; 1 μg/ml, Biolegend, USA), or HTR8/Svneo cells or DSCs for 48 h. In some wells, dCD8^+^ T cells (2 × 10^5^ cells) were plated in the upper chamber (0.4 mm pore size cell culture inserts, Millipore, Germany), while trophoblasts were plated in the lower chamber to establish indirect cell contact. Phorbol 12-myrstate 13-acetate (PMA) (50 ng/ml, Biolegend, USA), ionomycin (1 μg/ml, Biolegend, USA) and brefeldin A (10 mg/ml, BioLegend, USA), were added 4 h before the end of the 48 h culture for intracellular cytokine analysis. The cells were then harvested for flow cytometry analysis.

### Tim-3 and CTLA-4 blocking experiments

dCD8^+^ T cells were cultured (5 × 10^5^ per well) in the presence of anti-Tim-3 (10 μg/ml, clone F38-2E2, BioLegend, USA), anti-CTLA-4 (10 μg/ml, clone L3D10, BioLegend, USA), both of the two antibodies, or isotype control for 48 h. Brefeldin A, PMA, and ionomycin was added 4 h before the end of the culture. The cells were then collected for further analysis by flow cytometry.

### Mice

CBA/J female, DBA/2 male, and BALB/c male mice were purchased from Huafukang (Beijing, China) and maintained in an animal facility according to institutional and National Institutes of Health Guidelines. Eight-week-old CBA/J females were mated to DBA/2 males to establish abortion-prone models, and 8-week-old CBA/J females were mated to BALB/c males to induce normal pregnancy All the CBA/J females were inspected every morning for vaginal plugs. The day of visualization of a plug was designated as day 0.5 of pregnancy. Pregnant females received injections of anti-Tim-3 antibody (clone RMT3-23, BioLegend, USA), anti-CTLA-4 antibody (clone 9H10, BioLegend, USA), both antibodies, or isotype IgG i.p. at doses of 500, 250, and 250 mg on days 4.5, 6.5, and 8.5, respectively based on our previous publications^33,34^. All pregnant mice were monitored at day 10.5 of pregnancy. The percentage of fetal loss (the embryo absorption rate) was calculated as following: % of resorption = *R*/(*R* + *V*) × 100, where *R* represents the number of hemorrhagic implantation (sites of fetal loss) and *V* stands for the number of viable, surviving fetuses.

### Preparation of mouse cells

Uteri from pregnant mice were dissected free from the mesometrium and removed by cuts at the ovaries and cervix. The fetal and placental tissues were carefully removed and washed in PBS. Minced uteri were digested in RPMI 1640 supplemented with collagenase type IV and DNase I for 30 min at 37 °C with gentle agitation. Cells were cultured in RPMI 1640 supplemented with 10% FBS, 100 U/ml penicillin, 100 μg/ml streptomycin, and 1 μg/ml amphotericin B at 37 °C in 5% CO_2_ for 4 h to remove adherent stromal cells.

### Flow cytometry

Cell surface molecular expression and intracellular cytokine production were evaluated using flow cytometry. FITC-conjugated anti-human CD8, IFN-γ, Ki67, or anti-mouse CD8, PE-conjugated anti-human Tim-3, T-bet, TGF-β1, or anti-mouse T-bet or GATA-3, PE/CY7-conjugated anti-human IL-10, TNF-α, CD62L, CD8, IFN-γ, IL-17A, or TGF-β1, APC-conjugated anti-human CTLA-4, IL-4, Foxp3, ROR-γt, or anti-mouse TNF-α or IL-10, Brilliant Violet 421-conjugated anti-human CD107, Ki67, IL-4, IL-10, GATA-3, IL-17A, or anti-mouse IFN-γ or IL-4, Pacific blue-conjugated human CD44 (Biolegend, USA) antibodies were used. For intracellular staining, cells were fixed and permeabilized using the Fix/Perm kit (Biolegend, USA). Flow cytometry was performed on a Beckman-Coulter CyAn ADP cytometer (Beckman-Coulter, USA) and analyzed with FlowJo software (Tree Star, Ashland, USA).

### Statistical analysis

The statistical significance of differences between two groups was determined by the post hoc Dunnett *t* test. Multiple groups were analyzed with GraphPad Prism Version 5 by one-way or two-way ANOVA with Bonferroni post *t* tests. For all statistical tests, *p* values < 0.05 were considered statistically significant.

## Supplementary information

Supplementary figs

Supplementary figure legends
