# Supplementary figures and images for: The appropriate frequency and function of decidual Tim-3+CTLA-4+CD8+ T cells are important in maintaining normal pregnancy
Source: Cell Death Dis. 2019 May 28;10(6):407. doi: 10.1038/s41419-019-1642-x (PMC6538701; doi:10.1038/s41419-019-1642-x)

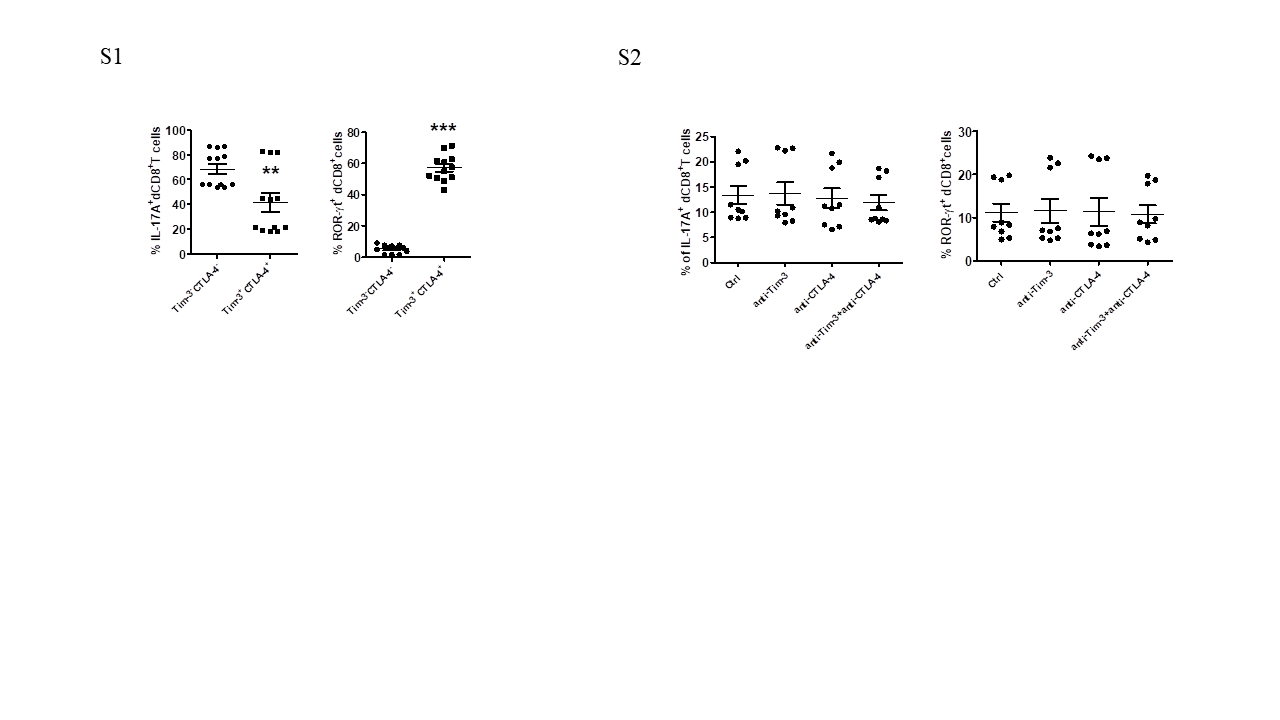

Supplement: Supplementary file 1 — Supplementary figs [file 41419_2019_1642_MOESM1_ESM.tif]
