# Supplementary material for: The appropriate frequency and function of decidual Tim-3+CTLA-4+CD8+ T cells are important in maintaining normal pregnancy
Source: Cell Death Dis. 2019 May 28;10(6):407. doi: 10.1038/s41419-019-1642-x (PMC6538701; doi:10.1038/s41419-019-1642-x)
Supplement: Supplementary file 2 — Supplementary figure legends [file 41419_2019_1642_MOESM2_ESM.docx]

**Figure S1.** Expression of IL-17A and ROR-γt in Tim-3^+^CTLA-4^+^, and Tim-3^-^CTLA-4^-^dCD8^+^ T cells from the first trimester of normal pregnancy. n=12. Data represent the mean ± SEM.

**Figure S2.**Expression of IL-17A and ROR-γt of dCD8^+^ T cells cultured for 48h in the presence or absence of anti-Tim-3 antibody (10 μg/ml), anti-CTLA-4 antibody (10 μg/ml), or both.
